# Synthetic Birdsongs as a Tool to Induce, and Iisten to, Replay Activity in Sleeping Birds

**DOI:** 10.3389/fnins.2021.647978

**Published:** 2021-07-05

**Authors:** Ana Amador, Gabriel B. Mindlin

**Affiliations:** ^1^Department of Physics, University of Buenos Aires, Buenos Aires, Argentina; ^2^IFIBA, CONICET, Buenos Aires, Argentina

**Keywords:** birdsong, dynamical systems, nonlinear dynamics, replay, acoustics

## Abstract

Birdsong is a complex vocal behavior, which emerges out of the interaction between a nervous system and a highly nonlinear vocal device, the syrinx. In this work we discuss how low dimensional dynamical systems, interpretable in terms of the biomechanics involved, are capable of synthesizing realistic songs. We review the experimental and conceptual steps that lead to the formulation of low dimensional dynamical systems for the song system and describe the tests that quantify their success. In particular, we show how to evaluate computational models by comparing the responses of highly selective neurons to the bird’s own song and to synthetic copies generated mathematically. Beyond testing the hypothesis behind the model’s construction, these low dimensional models allow designing precise stimuli in order to explore the sensorimotor integration of acoustic signals.

## Introduction

Birdsong is a behavior that emerges as a set of brain generated physiological instructions are delivered to respiratory and vocal muscles. These gestures, delicately synchronized, set in motion a variety of biomechanical processes that end up in the production of spectrally and temporally complex sounds. The vocal muscles control the configuration of the syrinx, the vocal organ, determining the tension of some tissues, whose oscillations modulate the airflow generating sound (Suthers et al., 1999; Suthers, 2001; Suthers and Margoliash, 2002). The nonlinear nature of the labial oscillations translates into their spectral content, and therefore into the timbre of the resulting sounds (Mindlin, 2017). In this way, the interaction between the nervous system and the nonlinear nature of the vocal organ determines the acoustic features of the uttered sounds (Mindlin and Laje, 2005; Amador and Mindlin, 2014). In fact, this simple description is the result of a long and rich interplay between experiments, theoretical models, and mathematical advances in the field of nonlinear dynamics. We will discuss some important moments of this rich history.

With this perspective, we explore the physics involved in birdsong production. Physics aims to provide minimal descriptions, i.e., the simplest mechanisms compatible with a given dynamics. But in a biological problem, it is not obvious which could be a criterium for deciding that a model is acceptable. The simplest option in biology may not be the most successful in an evolutionary context. This essential difference between biology and physics makes development of biomechanical models a delicate issue.

Birdsong is also thoroughly studied in the field of neuroscience as an animal model for vocal learning (Bolhuis and Gahr, 2006; Zeigler and Marler, 2012; Nieder and Mooney, 2020). Approximately 40% of the known bird species require the exposure to a tutor in order to develop their songs. The learned physiological gestures required to generate those complex vocalizations is achieved through a complex neural architecture that involve several neural nuclei. A remarkable characteristic of birdsong is the degree to which cortical areas contribute to the generation of vocal patterns. In songbirds, the neural nucleus HVC (used as proper name, analogous to a premotor cortex in mammals), has proven to be essential to the generation of learned vocalizations (Nottebohm et al., 1976). Since this nucleus also receives inputs from the auditory pathway, it was natural to explore the nature of its auditory response to a variety of stimuli (Janata and Margoliash, 1999; Amador and Margoliash, 2011). As a result of those explorations, it was found that some HVC neurons that present song premotor activity, also respond to playback of the bird’s own song (BOS). This response was found to be highly selective to BOS, i.e., responding more to BOS than to other acoustic stimuli as white noise, tones, and even conspecific songs (CON) or the BOS played in reverse (REV) which preserves the spectral content but affects the temporal information of the stimulus (Margoliash, 1983; Margoliash and Konishi, 1985). In many cases, the evoked activity through auditory stimulation and the premotor activity were very similar (Prather et al., 2008). Moreover, in anesthetized and sleeping birds, auditory stimulation with the BOS can entrain motor like activity in the whole network composed by the neural nuclei involved in birdsong production (Doupe and Konishi, 1991; Dave and Margoliash, 2000; Cardin and Schmidt, 2003). Moreover, recently it was shown that sleeping songbirds exposed to the recordings of their own songs display motor like activity patterns in their syringeal muscles (Bush et al., 2018). Syringeal muscles are innervated by motor neurons contained in the tracheosyringeal part of the hypoglossal motor nucleus (nXIIts), which is part of the song system neural network. Therefore, measurements in the syringeal muscles constitute a direct readout of the song system.

The high selectivity displayed by some neurons to the BOS allows to have a well-defined challenge: a physical model of the avian vocal organ should include enough elements so that the sound it generates is able to elicit responses in neurons highly selective to the BOS. It also paves the way to explore other important issues: is there a hierarchy of importance within the parameters that are included in our minimal models? How does the response degrade as the parameters of the model are varied? Beyond validating a physical model, the strategy of stimulating with physically realistic models allows varying interpretable parameters and exploring the way in which responses degrade.

Beyond the possibility of validating biomechanical hypothesis, and its use as generators of auditory stimuli, biomechanical models of birdsong production can be used to explore brain activity during sleep. It has been recently shown that spontaneous replay activity generated in a sleeping bird can be registered in syringeal muscles (Young et al., 2017). A code for translating brain activity into song is not yet available, but there have been significant advances in the association between acoustic features and certain patterns of muscle activity. In this work we will describe dynamical models of birdsong production and discuss the physics behind them. We will discuss their potential as generators of acoustic stimuli, and the possibility of using them to translate a bird’s neural activity during sleep into actual sound.

## Towards a Low-Dimensional Biomechanical Model

Evolutionary distant species as humans and songbirds produce some of their communication sounds through similar mechanisms, engaging the motor systems in charge of controlling respiration, the vocal organ and the upper vocal tract (Wild, 2004; Riede and Goller, 2010; Elemans, 2014). In the case of birdsong production, respiratory gestures are used to generate airflow, which induces connective tissue masses (known as labia) to oscillate (Goller and Larsen, 1997; Suthers et al., 1999). These oscillations bare some similarity with those displayed by the human vocal folds when voiced sounds (like vowels) are produced (Titze, 1994; Mindlin and Laje, 2005).

This brief description does not honor the conceptual and experimental difficulties in reaching this picture. When a human wants to imitate a bird, the person whistles. It is therefore natural to conjecture that at least for some bird species, some form of whistle-like mechanism could be responsible for the generation of at least the tonal sounds. And in fact, in one of the earliest papers on the subject of birdsong production, the anatomist George Cuvier proposed in 1800 an analogy between a trombone and the song system (Cuvier, 1800). Although in bronze instruments the sound source is the oscillation of the lips, it is the strong feedback of the instrument what stabilizes the sounds source (Adachi and Sato, 1996). This is also the case in a whistle, where the sound source is the periodic vortex detachment from a jet. In both cases, the changes in the resonances of the device attached to the source would determine the fundamental frequencies of the sound (Olson, 1967). A conceptual breakthrough would be provided by Thorpe (Thorpe, 1959), who in 1959 suggested that a more appropriate analogy for the song system would be found in human phonation. The human model is a strong conceptual departure from the previous ones, since independently of the nature of the sound source dynamics (vortex departure or tissue modulations of an airflow), its frequency is unaffected by the properties of the attached cavity. In human phonation, tongue, lips and jaw determine the way in which the spectral richness of the signal generated by the sound source is filtered, but they do not condition the dynamics of the vocal folds (Titze, 1994). An additional conceptual challenge to the models that required a strong interaction between a tract and a sound source was provided by the observation of sounds built out of two non-harmonically related tones. It was Greenewalt (Greenewalt, 1968) who observed that two sound sources could not act independently of each other if their dynamics was conditioned by a unique tube. This delicate observation of the spectral content of some birds’ songs could be possible before the development of computational tools as the spectrograph. But beyond this smart observation, experimental evidence was necessary. A crucial experiment was carried out by Nowicki (Nowicki, 1987), who recorded the songs and calls of nine species of oscine birds in a Helium atmosphere. No change was found in the values of the fundamental frequencies, although the speed of sound in this altered atmosphere changes significantly. This built confidence in the theory that the sound source’s dynamics is basically independent of the resonances of the attached tract. Notice that when a human speaks after the inhalation of Helium, the perceived change in pitch if due to the alteration of the filter: the fundamental frequencies are basically unchanged (Nowicki and Marler, 1988).

At this point, the idea of sound sources capable of displaying dynamics largely independent of the tract was well established. But the general view was that the medial tympaniform membranes were the principal sound generators. Goller and Larsen performed a series of experiments surgically incapacitating the tympaniform membranes as vibratory sources in cardinals (*Cardinalis cardinalis*) and zebra finches (*Taeniopygia guttata*), which were yet capable of singing nearly normal songs (Goller and Larsen, 1997). Moreover, they studied the syrinx filming it endoscopically during spontaneous vocalizations and were capable of describing the reconfiguration that the syrinx undergoes in the process. They found that the vocalization starts with a rostral movement that stretches the syrinx and pushes the medial and lateral labia into the bronchial lumen. They also reported that during song, these tissues oscillate (although at the time, the resolution of the film did not allow them to directly compare the labial frequency with the song’s fundamental frequency). With this experimental evidence at hand, it was clear that there were many common elements between the mechanisms involved in birdsong production and the generation of some of the sounds that are used in human vocalizations. In particular with those called voiced sounds, that are generated when the airflow sets in motion the vocal folds (Titze, 1994).

In the case of the human voice, the search for a computational model implementing the acoustic principles of sound production was motivated by the need to obtain speech synthesis by machines. In Bell labs Flanagan and Ishizaka developed the first finite element computational implementation of a mathematical model of voiced sounds production (Ishizaka and Flanagan, 1972). They assumed that the vocal cords could be approximated by a self-oscillating sound source built out of two stiffness-coupled masses. The tract was modeled as a transmission line. A one-dimensional Bernoulli flow was used to model the dynamics of the air between the masses, and a plane wave was used to approximate the behavior of the fluid in the tract. These basic elements are present in many models of voice production until these days. This model allows a mathematical description of the problem in terms of a dynamical system, i.e., a finite set of ordinary differential equations ruling the dynamics of a finite set of variables. In particular, the positions of the masses will obey Newton’s laws. The forces acting on each mass include the Bernoulli force, which requires computing the pressure of the air at the lumen, the elastic forces that mimic the internal elastic properties of the vocal folds, and the highly nonlinear dissipation that accounts for the energy loses that occur when the folds bump into each other. With its complexity, this is a finite set of ordinary differential equations, which is much easier to study than a continuous model, expressed by a nonlinear partial differential equation. Still, this model can display a remarkable variety of qualitatively different solutions. The dynamical model studied by Herzel et al. (1995) allowed them to account for several irregularities found in the voices of patients with pathological conditions. This model, with its nonlinearities, and its dimensionality, is capable of displaying not only periodic solutions (what would be enough to reproduce a simple sound), but also quasiperiodic and chaotic solutions, whose spectra are extremely rich.

Yet, the simulations of these 2-mass models (the name is derived from the two masses used to represent each vocal fold) showed that for wide regions of the parameter space, the four masses representing the two vocal folds would oscillate periodically, with fixed phase differences between them. Interestingly enough, this collective dynamic can be displayed even for some degree of asymmetry between the opposing folds (Steinecke and Herzel, 1995). More importantly, it suggested the possibility of describing this behavior in terms of simpler spatial modes, and therefore, with a smaller number of equations. Titze carried out this additional simplification in the models for voice production (Titze, 1988). He observed that the phase difference between the upper and lower mass in representing each of the folds could be interpreted as a unique flapping surface wave. At this point bird’s labia and human vocal folds can be treated similarly. In the following section, we will discuss the implementation of this model in greater detail.

Once a sound is generated by self-sustained oscillations (of vocal folds in the human case, of syringeal labia in the case of birds), the sound is passively filtered. In the case of human communication, the delicate articulation of motor gestures controlling lips, tongue and jaw allows the generation of different vowels, and it is the case for many human languages that many of those sounds are generated with a modest variation of the fundamental frequency during normal speech (Titze, 1994). Turns out that humans are not the only vertebrates with tunable vocal tract filters. It was shown (Riede et al., 2006) that birdsong is accompanied by cyclical movements of the hyoid skeleton and changes at the cranial end of the esophagus that allow the tuning of the vocal filters to enhance the song’s fundamental frequency.

## A Mathematical Implementation of a Flapping Model of the Sound Source

As discussed in the previous section, an important reduction in the dimensionality of the problem is to consider the oscillating labia (in principle with many degrees of freedom) as an oscillating mass subject to the action of forces. To define a dynamical model for this mass, the Newton’s second law is used, which states that the sum of forces $\sum_{i}F_{i}$ acting on a body is equal to the mass *m* of the body multiplied by the acceleration of its center of mass *a* : $m\,a=\sum_{i}F_{i}$. In this way, the equation of motion is defined by the following differential equations with variable *x* representing the medial position of a labium measured from its equilibrium position:

(1) $$\frac{d\,x}{d\,t}=y$$

(2) $$m\,a=m\,\frac{d\,y}{d\,t}=-k\,x-b\,y+f_{0}+F_{d}\,\left(x,y\right)+G\,\left(p_{s\,u\,b}\,x,y\right),$$

with −*kx* the elastic force, −*by* the dissipative force, *f*_*0*_ a constant force generated by the syringeal muscles. The function *F*_*d*_(*x*,*y*) = −*cx*^2^*y* represents a nonlinear dissipative force that depends on the labium position, and *G*(*p*_*sub*_*x*,*y*) represents the force due to the airflow. For sufficiently high values of the pressure’s airflow the labia start to oscillate, with a wavelike upwards motion (Goller and Larsen, 1997; Elemans et al., 2015). This wave can be described in terms of two basic modes: a lateral displacement of the labia, and a flapping-like motion, leading to an out-of-phase oscillation of the top and bottom portion of the labia [for a detailed description see Titze (1988, 1994); Gardner et al. (2001); Mindlin and Laje (2005)]. In this way, a specific function for *G*(*p*_*sub*_*x*,*y*)=*p*_*sub*_*a*_*lab*_*f*(*x*,*y*), is defined, being *a*_*lab*_ the lateral labial area and *f*(*x*,*y*) a function including geometrical aspects of the labia and its flapping motion. The dynamical model of the sound source is completed by assuming labial nonlinear restitution elastic properties and nonlinear dissipation, with *k* = *k*(*x*)=*k*_1_ + *k*_2_*x*^2^ and *b* = *b*(*y*)=*b*_1_ + *b*_2_*y*^2^:

(3) $$\frac{d\,x}{d\,t}=y$$

(4) $$m\,\frac{d\,y}{d\,t}=-k_{1}\,x-k_{2}\,x^{3}-b\,\left(y\right)\,y+f_{0}-c\,x^{2}\,y+p_{s\,u\,b}\,a_{l\,a\,b}\,f\,\left(x,y\right)$$

**FIGURE 1:**
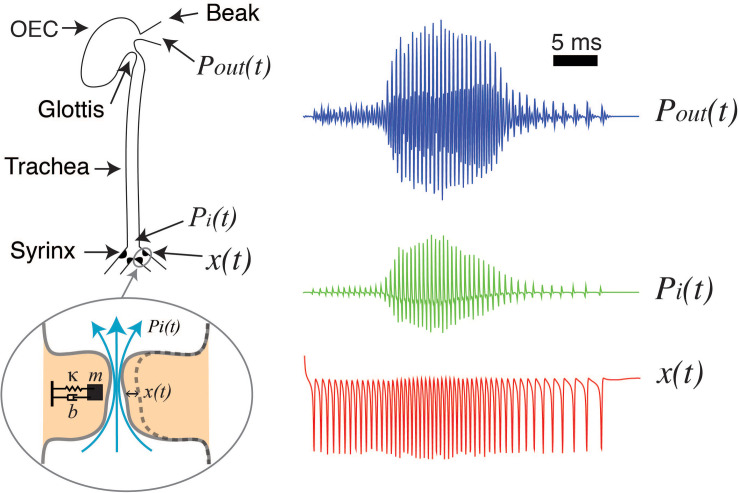
Schematized view of the sound source and vocal tract of songbirds, and its filtering effects. The syringeal membrane modeled as a mass (*m*) with damping (*b*) and a restitution force (*k*), is able to display auto-sustained oscillations when nonlinear functions representing a flapping movement of the labia are introduced. These oscillations are the sound source [x(t), red line, representing the medial position of a labium] as pressure variations are generated at the input of the trachea [P_*i*_(t), green line], and then filtered by the oro-esopharingeal cavity (OEC). The output of the dynamical model is the pressure [P_*out*_(t), blue line] that will result in the synthetic sound (see text for further details). Adapted from Amador et al. (2013).

For a detailed deduction of these equations and further details see (Titze, 1988; Gardner et al., 2001; Mindlin and Laje, 2005; Amador and Mindlin, 2008; Mindlin, 2017). With the appropriate parameter values, an energy transfer from the airflow to the labia allows the emergence of auto-sustained oscillations during phonation. In this way, a low dimensional model can account for the labia oscillations during birdsong production. To complete the model of phonation, the vocal tract needs to be included. The sound source is the rate of mass injection for unit of volume $s\,\left(t\right)=\frac{\partial q}{\partial t}$, being *q* proportional to the air velocity (Howe, 2003). These density perturbations, plus the back propagating wave, build the pressure fluctuations at the input of the trachea *p*_*i*_(*t*) and the transmitted pressure wave excites the oro-esopharingeal cavity [OEC, see Fletcher et al. (2006); Perl et al. (2012)]. The OEC is modeled as a Helmholtz resonator (Perl et al., 2011). These steps are illustrated in Figure 1, that displays a schematic of the syrinx, the trachea and the OEC. The different time traces at the right of Figure 1 show the labial motion (bottom, red), the pressure at the input of the tract (middle, green) and the output pressure (top, blue).

It is pertinent to discuss how much of the synthesized acoustics will depend on the specific hypothesis used to build the model. For example, the specific nonlinearity used to represent the nonlinear dissipation. In this problem, we are particularly interested in the transition from stationary to oscillatory labia, as this is the transition from silence to sound. This qualitative change in the dynamics is known as a bifurcation (Strogatz, 1994). Physical models, for parameter values in the vicinity of a bifurcation can be taken to their “normal forms.” These are minimal dynamical systems capturing the basic bifurcations (Guckenheimer and Holmes, 1997). It is one of the most beautiful results in nonlinear dynamics that for a given dimensionality, the qualitatively different number of ways in which an oscillation can be born is small, and therefore many nonlinear dynamical systems which look very different (i.e., contain different nonlinear terms), can be taken to the same normal form by the appropriate change of variables.

**FIGURE 2:**
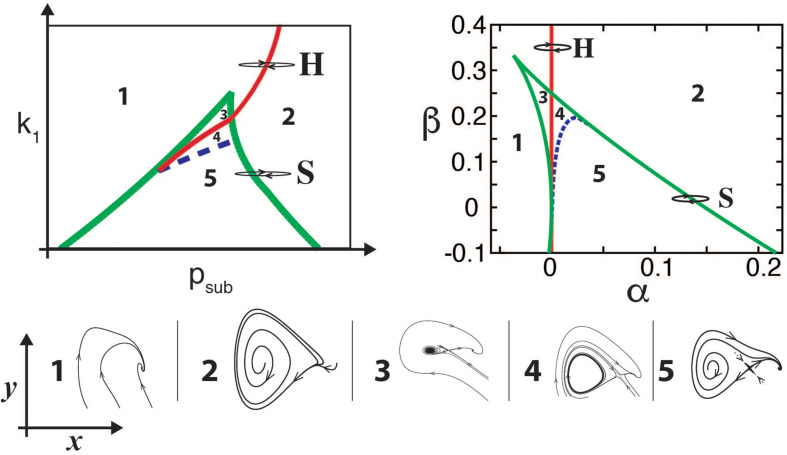
Bifurcation diagrams of birdsong production models. For the physical model (upper left panel), the diagram is displayed in the (*p*_*sub*_, k_1_) parameter space, representing the subglottal pressure and the labial tension, respectively. The bifurcation diagram of the normal form (upper right panel) is displayed in the (α,β) parameter space. Typical portraits of the (*x*,*y*) phase spaces for each region are shown in the corresponding numbered lower insets, showing the different dynamical regimes that occur in each region separated by bifurcation lines. In both upper panels, continuous red line represents a Hopf bifurcation, dashed blue line the homoclinic bifurcation, and green lines the saddle-node bifurcations. Paths H and S in the parameter space represent different ways to start oscillations of the labia, either by crossing the Hopf (H) or the saddle-node in limit cycle (S) bifurcation lines, thus generating either tonal or spectrally rich sounds. Adapted from Sitt et al. (2010).

It turns out that in many cases, identifying the underlying normal form of a model is enough to capture the timbre of the synthesized sounds. For example, oscillations born in Hopf bifurcations lead to tonal sounds, while those born in “Saddle Node in Limit Cycle” (SNILC) bifurcations are spectrally rich, giving rise to rougher sounds (Amador and Mindlin, 2008; Sitt et al., 2008). Both these bifurcations can be found for parameters close to those where a Hopf line is tangent to a saddle node curve (see bifurcation diagram of the model in Figure 2, left). This is known as a “Takens–Bogdanov” bifurcation, and scaling the time through a constant γ the normal form for this bifurcation can be written as:

(5) $$\frac{d\,x}{d\,t}=y$$

(6) $$\frac{d\,y}{d\,t}=-\alpha \,\gamma^{2}-\beta \,\gamma^{2}\,x-\gamma^{2}\,x^{3}-\gamma \,x^{2}\,y+\gamma^{2}\,x^{2}-\gamma \,x\,y$$

where α and β stand for the unfolding parameters [for a detailed description see, for example (Sitt et al., 2010; Perl et al., 2011; Mindlin, 2017)]. The bifurcation diagram of this dynamical system is shown in Figure 2, upper right panel. In other words, for parameter values close to those in which the physical model presents a Takens–Bogdanov bifurcation, a nonlinear change of variables will allow us to write it in this simpler way. The bifurcation diagrams of the complete model (Eqs. 3, 4) and its normal form (Eqs. 5, 6) shown in Figure 2 highlight the important normal form feature of preserving the dynamics of the complete model. Given this similarity, the parameter α is associated with the subglottal pressure (*p*_*sub*_) and the parameter β with the labial tension (*k*_1_).

The generation of sound further requires the input of physiological instructions, or simple time dependent parameters (α(*t*), β(*t*)) that guarantee that the synthesized sounds present acoustic features similar to the bird’s song (Perl et al., 2011). The interesting aspect of this problem is that the nonlinear nature of the model, and more specifically, the bifurcations taking place, guarantee that given a fundamental frequency, the spectral content of the synthesized signal will be correct (Sitt et al., 2008). In other words, the spectral content is provided by the dynamics.

Normal form reduction is the ultimate physics exercise: not only considering the minimal number of effects when formulating a model, but further simplifying the system of equations by eliminating, through nonlinear changes of coordinates, nonlinear terms. In the example shown here, it also reduces substantially the number of physiological related parameters. Can such a reduced, simplified system constitute a reasonable model for a bird’s song?

## Inducing Physiological Responses With Low-Dimensional Models

In order to process and establish vocal communication, it is important to find a correspondence between the sensory and motor codes used to represent and generate a given signal. One plausible way to establish their correspondence is to count with neurons that are active both when the animal perceives the communicating signal, and when it produces it. Neurons in HVC highly selective to the BOS respond in this way (Prather et al., 2008). We will not discuss here the plausible role that these neurons might play in the process of learning. Instead, we will show how these neurons can be used as tools to explore the bird’s response to a variety of synthetic stimuli generated by computational models based on the physics of birdsong production.

The high selectivity of HVC neurons to the BOS is itself a sensible way to test whether a normal form, which is a simplified system of nonlinear equations derived from of an already idealized physical model, would be capable of generating meaningful auditory stimuli. This would pave the way to explore the degradation of the response as different parameters present in the model are varied.

**FIGURE 3:**
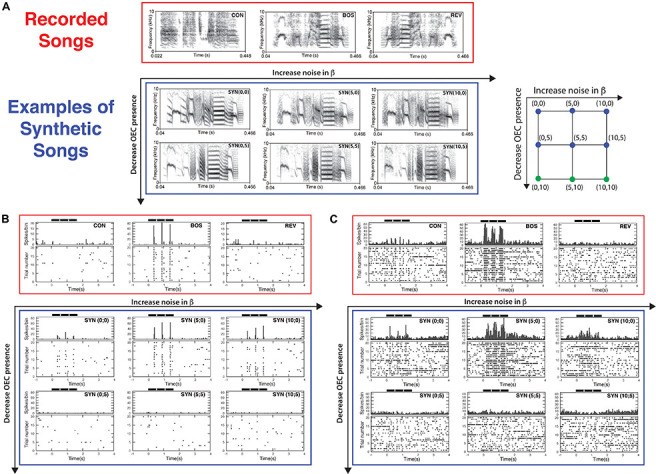
Testing a low-dimensional model of vocal production and the relative importance of physiological-related parameters of the model. **(A)** Spectrograms of the recorded songs and synthetic songs used as auditory stimuli presented to sleeping birds. The recorded songs (BOS, REV, and CON) were used to test the neurons selectivity to BOS. A series of synthetic variants were generated (SYN, typically 9–12) using parameters from a grid. The indexes (a, b) in the legend SYN (a; b) refer to the level of noise in β and dissipation of the OEC resonator, respectively. These synthetic songs variants are represented as blue dots in the grid of the right panel. **(B)** Example of a raster plot and histogram of a phasic neuron response to the presentation of the auditory stimuli shown in **(A)**. The timing of the three repeated motifs that were presented is indicated by the bold horizontal lines. **(C)** Same as **(B)** but showing a recording of a tonic neuron. Adapted from Amador et al. (2013).

An example of the selectivity property of HVC neurons is shown in Figure 3 (red boxes). Figure 3A shows the spectrograms of all the auditory stimuli presented to the bird. In Figure 3A the red box highlights the stimuli used to test the neural selectivity to bird’s own song (BOS). This is a well-established protocol including the auditory stimulus to be tested (BOS) and two control stimuli: the bird’s own song played in reversed (REV) and a conspecific song, i.e., song from a different bird of the same species (CON). Notice that REV has all the same sounds that BOS has but in a reversed order and CON contains similar sounds of the species but each individual has its own song due to the learning process to acquire it. Figures 3B,C show the neural responses to the presented stimulus: each dot in the raster plot indicates the time when a spike occurs. In the example shown here, 20 trials of each stimulus were presented (shown as black bars at the top of each panel) and each row in the figure shows the spike occurrence along each trial. To have a graphical quantification of the neural response, a histogram of the occurred spikes is shown in the upper panel. A robust response to BOS and no significant responses to REV and CON is a signature of the selective property of the recorded neuron (Figures 3B,C, red boxes). HVC selective neurons may exhibit phasic responses (Figure 3B) or tonic responses (Figure 3C) to the stimuli presented.

This kind of neural responses suggested that in order to generate a significant neural response the auditory stimulus should be very similar to BOS. To generate the synthetic copy of the bird’s own song (SYN), the fundamental frequencies of the recorded bird’s own song were computed as a function of time. For each frequency in the time series, the parameter α was computed to generate a time trace with that fundamental frequency. The interesting contribution of the normal form is that the spectral content of the signal is determined by the distance of the parameter α to the bifurcation line where the oscillations are born. Therefore, the timbre of the sound is severely conditioned by its fundamental frequency.

**FIGURE 4:**
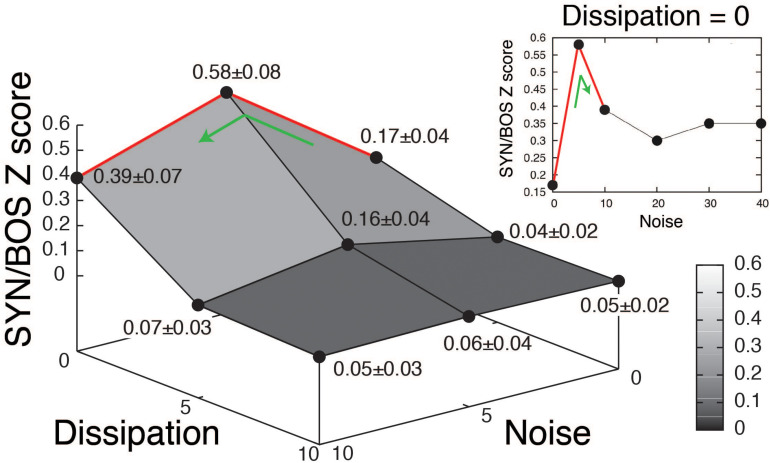
Quantification of neural response to synthetic songs variants. Grouped data of 30 recorded neurons in HVC selective to the bird’s own song (BOS). The synthetic songs (SYN) variants changing the level of noise in β and dissipation of the resonator representing the OEC are organized as in the grid shown in Figure 3. The Z scores of SYN, were normalized to the BOS Z score, and averages across neurons were reported as means of normalized responses ± SEM. The inset (Dissipation = 0) shows the neural responses to high levels of noise with full presence of the OEC resonator. Adapted from Amador et al. (2013).

A grid of stimuli can be generated by smoothly changing parameters in the model. In this way, it is possible to explore their relative contribution to the elicitation of selective responses. An example of such an exploration is shown in Figure 3 (blue boxes). The level of noise in the parameter β, as well as the dissipation of the resonator representing the OEC were varied. A high value for the dissipation corresponds to decreasing the OEC presence in the model, therefore decreasing the resonator effect, which is enhancing a specific frequency band of the sound signal. For each set of parameters, a synthetic song can be generated, using it to acoustically stimulate the bird, while recording the response of a selective HVC neuron. When the level of noise was set to zero and the dissipation was high no significant responses were found in HVC selective neurons. Notice that when the parameters are modified the synthetic copies can be very similar to BOS but the neural responses can change substantially [see spectrograms of SYN(0;0), SYN(5;0), SYN(10;0) in Figure 3A and its neural responses in Figures 3B,C]. The neural responses to a grid of SYN stimuli with identical timing [same functions for (α(*t*), β(*t*))] but different spectra from BOS identified optimal estimates for two remaining free static parameters. In this way, SYN(5;0) can be selected as the best fit according to the neural responses obtained. Notice that the timing and shape of the histograms are very similar between SYN(5;0) and BOS but the responses to SYN(5;0) show a reduced amplitude. A quantification of this can be achieved by normalizing the neural response to SYN with the neural response to BOS. The grouped data of this quantification is shown in Figure 4 (Amador et al., 2013). A maximum for the neural response is clearly noticeable, corresponding to an intermediate level of noise and full presence of OEC resonator (zero dissipation), i.e. SYN(5;0). Notice that the maximum level of response is 58%, showing that there are some acoustic features of BOS that the synthetic copies are not yet able to reproduce. It is also interesting to notice that the neural response decreases substantially for synthetic acoustic stimuli with no noise, but it is rather robust to high levels of noise (see inset of Figure 4).

**FIGURE 5:**
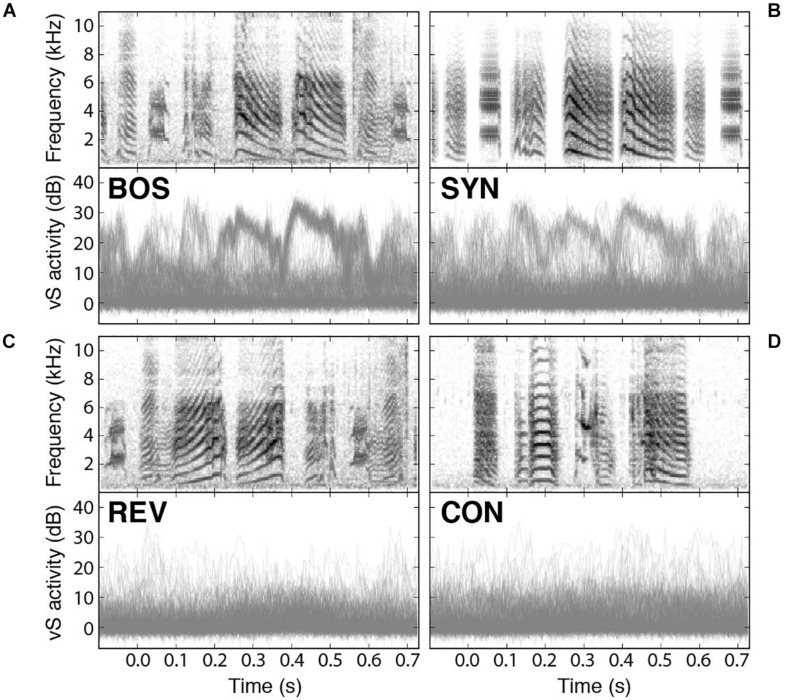
Syringeal muscle activity is selectively evoked by auditory playbacks. Playback responses in sleeping zebra finches to **(A)** the bird’s own song (BOS), **(B)** a synthetic copy of the bird’s own song (SYN), **(C)** the bird’s own song played in reverse (REV), and **(D)** a conspecific song (CON). In all cases, the top panel shows the spectrogram of the auditory stimulus presented, and the bottom panel shows an overlay of all recorded vS responses to those songs. Adapted from Bush et al., 2018.

Recently, it has been reported that spontaneous replay during sleep could be detected in syringeal muscles (Young et al., 2017). Also, muscle activity can be elicited when the sleeping bird was acoustically stimulated with presentation of BOS and synthetic copies of the song (SYN) (Bush et al., 2018). In fact, electromyograms (EMGs) of a syringeal muscle show playback-evoked patterns strikingly similar to those recorded during birdsong production. Interestingly enough, the activity of the muscle integrates instructions from many neurons, and therefore provides a reading of global nature of the selective activity in the bird’s brain. Therefore, it is possible to read a global signal with the potential to provide detailed aspects of the responses to the different stimuli. As we can see in Figures 5A,B, the response to the BOS is very similar to the response to the synthetic stimulus (SYN). The top panels of Figure 5 display the spectrograms of the presented auditory stimuli while the bottom ones display an overlay of the recorded responses in the syringealis ventralis (vS) muscle of zebra finches during sleep. There is a weaker response to the presentation of SYN but notice that the reduction is in the number of elicited responses for the synthetic stimuli, preserving the shape of the response. Figures 5C,D show the response to the temporal reversed bird’s own song (REV) and to a conspecific song (CON), respectively, highlighting the selectivity to BOS of these responses.

**FIGURE 6:**
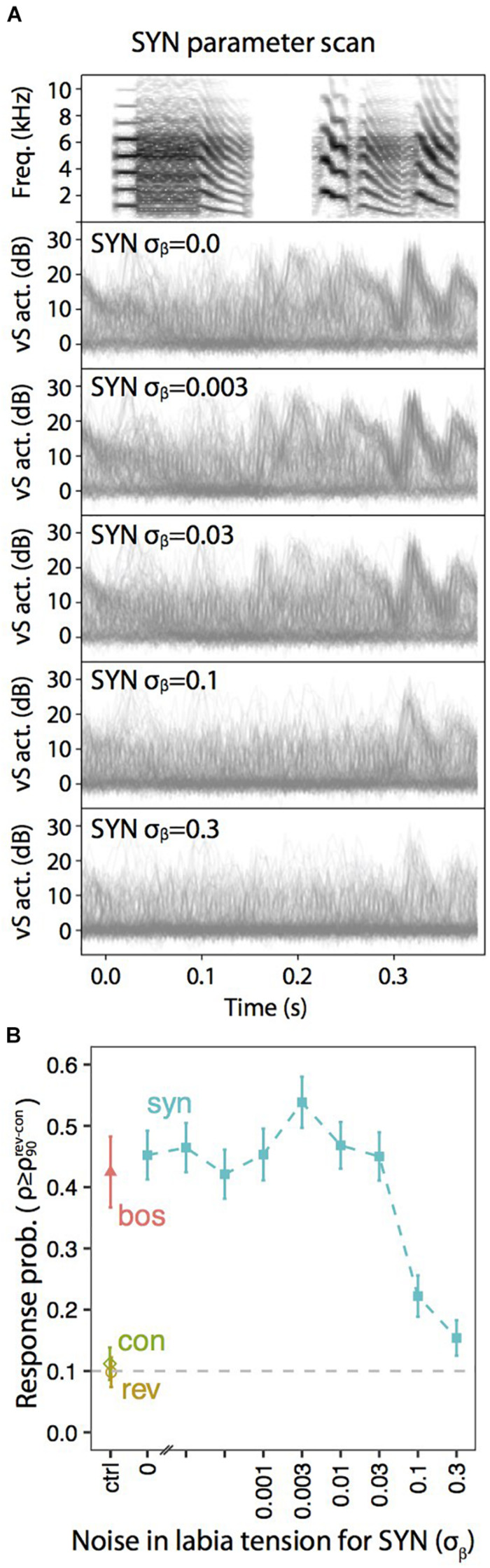
Degraded auditory stimuli evoke motor gestures with lower probability. **(A)** Overlays of vS activity traces elicited by motifs synthesized with the indicated amount of noise (σ_β_) added to the parameter representing the tension of the labia, β(*t*). **(B)** Response probability, calculated as the fraction of playbacks with normalized projection score (ρ) higher than the 90th percentile of REV and CON ($\rho_{90}^{R\,E\,V-C\,O\,N}$). Adapted from Bush et al. (2018).

A similar exploration in the static parameters that was tested with neural responses (Figures 3, 4) can be performed with muscle responses. Figure 6 shows the results obtained when a sleeping bird was subjected to a variety of acoustic stimuli, while the activity of the syringealis ventralis muscle (vS) was monitored. The different synthetic stimuli were generated varying the level of noise added to the physiological parameters representing the labial tension. In the second-sixth panels of Figure 6A, we show the overlays of vS activity when the noise is increased, for a synthetic song whose spectrogram is displayed in the first panel. Figure 6B shows the response probability as a function of the noise. As the noise is increased, there is a region where the responses remain high and even increase, and at some point, when the noise is very high, the vS response gets smaller. When the response probability is reduced, there are some presentations of the synthetic stimulus that elicit muscle responses. Remarkably, in these cases, the elicited vS activity patterns match execution patterns in shape and timing, indicating an all-or-nothing activation of the vocal motor program [see (Bush et al., 2018) for further details]. This is interesting, since continuously degraded songs could drive a response in an all or none fashion, consistent with an attractor dynamics (Amit, 1992; Mooney, 2020). If the auditory stimulus was close enough to the BOS, as to fall into the attractor’s basin, the system would respond very similarly to the way it would to the BOS. Otherwise, the system would simply fail to fall to the attractor.

Notice that the results presented in Figures 5, 6 for syringeal muscle responses to acoustic stimuli are similar to the results presented in Figures 3, 4 for HVC neural responses.

## Questions Suggested by These Physiological Responses

Obtaining responses to synthetic stimuli from highly selective neurons is an interesting result in itself. A physical model, and specifically the one described in detail in this review, assumes a number of non-trivial assumptions: reduced number of active modes in the labial dynamics; synchronized movement of opposing labia; a nonlinear dissipation; a nonlinear restitution force; a simplified computation of the inter labial pressure, among others. Such a model needs to be capable of displaying a bifurcation from quiescent to oscillatory labia capable of providing a spectral richness which, when properly filtered by a resonator, is realistic enough to elicit the measured physiological responses. Previous work indicated that it is not only the spectral richness of some sounds, but the way the spectral content correlates with frequency what constitutes the fingerprint of the bifurcation (Sitt et al., 2008; Mindlin, 2017). From that perspective, the simplification of the physical model to the normal form should not be considered as an additional simplification, but as the reason itself the model was successful.

The responses obtained when the static parameters are varied, are very informative as well. A variation of the dissipation of the Helmholtz resonator used to model the OEC gave rise to a variation of the neural response. A study performed in Northern cardinals (*C. cardinalis*) showed that the OEC volume could be modified during song production so that the resonant frequency of the resonator matches the fundamental frequency of the sound (Riede et al., 2006). In other words, it was shown that the birds have direct control of the OEC to enhance the tonal sounds decreasing the energy in the higher harmonics (Fletcher et al., 2006). In zebra finches though, some vocalizations have fundamental frequencies that are an order of magnitude smaller than the typical resonant frequency of the OEC. In fact, the low frequency sounds used in zebra finch song are spectrally very rich, with the OEC enhancing reasonably high harmonics of those sounds. A similar phenomenon is found in a South American suboscine (*Phytotoma rutila*), where the combination of a spectrally rich sound source filtered by a cavity of a much higher resonant frequency allowed to predict the precise way in which body size is encoded in its vocalizations (Uribarri et al., 2020). For the sound production mechanism that consists of a pulsatile excitation of a static cavity, it can be expected a reliable signature of body size in the peak frequency. Although there is no direct evidence that conveying size information is a relevant feature in zebra finch vocal communication, the works discussed here show that 1. the vocal production mechanism allows the sound to carry size information of the individual generating the sound, and 2. that selective neurons in the song system are very sensitive to changes of the parameters characterizing OEC size used in the generation of synthetic stimuli. These results can be then translated to syringeal muscle activity.

Fitting the fundamental frequencies of the sounds and incorporating the proper filters was not enough to elicit strong responses by selective neurons [see responses to synthetic stimulus SYN(0;0) in Figures 4B,C]. To maximize the neural response, noise to the parameter representing the labial tension needed to be added. Figure 4 shows the simultaneous variation of the noise level in β(t) and a parameter characterizing the dissipation of the OEC. An optimal noise level can be deduced from the existence of a maximum in the neural response [for the synthetic stimulus SYN(5;0)]. The tension of the labia is the result of forces exerted by syringeal muscles. In fact, the force emerges as the sum of several twitches that are the result of neural impulses sent by the nervous system. It is likely that for small muscles, the average does not add up to a smooth, continuous gesture. Therefore, a realistic time dependent physiological instruction has to present some level of roughness if it is used to synthesize a stimulus capable of eliciting a neural response (Amador et al., 2013).

## From Syringeal Muscle Activity to Sound

An interesting aspect of the replay recorded in the syringeal muscles is that using those physiological recordings, a reasonably link between muscle activity and behavior can be achieved. This cannot be done with neural activity recorded from a unique area of the song system, as the code used to represent behavior is yet to be unveiled.

In a previous section we described the syringeal muscle activity that was induced when a sleeping bird was subjected to stimuli similar to the BOS. In recent years, it was also reported that patterns of syringeal activity could spontaneously emerge (Young et al., 2017), similarly to the spontaneous neural replays previously reported (Dave and Margoliash, 2000). The global nature of the syringeal readout may allow to quantify the nature of those spontaneous replays. In zebra finches, it was reported that less than ten percent of the spontaneous replays could be classified as the complete motif used by the bird for singing, while close to fifteen percent of the replays included vS patterns that cannot be recognized as part of the motor gestures used for singing (Young et al., 2017). It is a song-like activity, noticeably different from noise, but which does not correlate with actual motor gestures. This raises the question of whether models can be used as a tool to “listen” to synthetic sounds associated to them, i.e., use the periphery as a window to explore the brain.

Unfortunately, only modest approximations to the actual songs could be synthesized at this point. All the syringeal muscles are necessary to properly set the vocal organ into the configuration necessary to synthesize a given sound, and measuring them all simultaneously is difficult. Dorsal muscles are likely to participate in the gating of the airflow (Goller and Suthers, 1996b), just as the syringealis ventralis muscle (vS) has been shown to participate in the modulation of the frequencies (Goller and Suthers, 1996a). On the other hand, some important pieces of the puzzle are already in place. In fact, it has been shown that from EMGs measurements in the vS muscle, it is possible to estimate the frequency modulations of zebra finch song. This requires transducing EMGs into muscle force by means of a model for the muscle, and this force into labial tension (Doppler et al., 2018). In order to build this bridge between EMG and frequency modulation several quantitative tools had to be developed. One of them is a model capable of translating EMG activity recorded in a muscle into the force that this can exert. Then another model is needed to account for the increase of tension that a labium can experience as force is applied to it. Since the restitution properties of a labium depend on this tension, the models that we discussed in the previous section will allow us to translate the changes of labial tension into frequency modulations.

Actually, this is an example of a much wider problem. The neural system does not work in isolation: it interacts with the physical world. It receives sensory inputs and generates outputs via muscles. Therefore, the activity and coding of the neural circuits can only be fully understood by considering the biomechanics of muscles, the body they are embedded, and the conditions of the exterior world (Tytell et al., 2011). For that reason, there is a rich history of modeling efforts in order to build these bridges (Keener and Sneyd, 1998).

**FIGURE 7:**
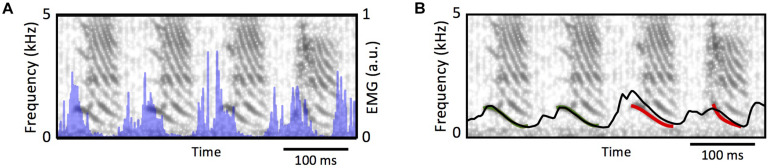
From syringeal muscle activity to acoustic features. **(A)** The spectrogram of a zebra finch song, and the simultaneous recording of the EMG at the syringealis ventralis muscle (blue traces). **(B)**, the proxy of the fundamental frequency, as the models in Doppler et al. (2018) are integrated. The red segment was used to tune the parameters, and the black line shows the model’s prediction of the fundamental frequency for the complete song. Adapted from Doppler et al. (2018).

From a phenomenological perspective, the muscle can be modeled as a sliding slack in parallel with a dissipative component, with the slack’s length is controlled by the electrical activity in the muscle (Shapiro and Kenyon, 2000). The labium can be modeled through a stretching viscoelastic element subjected to the force exerted by the muscle. In this way, as the electrical activity shortens the slack, the difference between the muscles actual length and the slack is transduced into force (Shapiro and Kenyon, 2000) through a nonlinear (exponential) function. These models were developed and implemented in Doppler et al. (2018). Figure 7 shows the spectrogram of a song, and the temporal evolution of the estimated fundamental frequency after integrating the muscle model and the labial model with the syringeal EMG as input (shown in blue in Figure 7A). Notice that during the temporal periods in which there is phonation, the models provide a good estimation for the fundamental frequency. Therefore, it is possible to find the parameters of the model for birdsong production and synthesize a good proxy for the song (see Figure 7B). Unfortunately, the information from that muscle cannot be used to also estimate the onset and offset times of the sound. Listening to dreams will require at least to measure the simultaneous activity of ventral and dorsal muscles to properly select from the synthetic sounds, the segments that will approximate the actual syllables.

## Discussion

Behavior emerges from the interaction of nervous systems, physical effectors and the environment. In this review we discuss how some subtle acoustic properties of birdsong are conditioned by the dynamics of the periphery. We also show how a dynamical model for the sound source can condition the acoustic properties of the generated sounds, specifically showing how different bifurcations may lead to spectrally different sounds.

This is not unique to birdsong. A recent work showed that the qualitative changes that occur in the vocalizations of marmosets during development are rooted in the nonlinear interaction between the nervous system and the biomechanics involved in respiration (Zhang and Ghazanfar, 2018). Moreover, analyzing the qualitatively different dynamics of a computational model for the phonatory system, they were capable of identifying parameters that allowed to mimic growth reversal and recover lost vocalizations. This integrative view has a longer history in other biological systems like locomotion, in which neural circuits generate patterns that are coupled to the environment by the body–limb system, and the importance of the biomechanics involved is very clear (Tytell et al., 2011). But in other fields, this integrative approach is yet to be embraced. Across vertebrates, progressive changes in vocal behavior during postnatal development are mostly analyzed from the development of neural circuits. How the changing body influences vocal behavior is only recently being integrated into the analysis (Zhang et al., 2019).

In this work we have reviewed an interactive journey between modeling and experiments in the study of birdsong production. The response of highly selective neurons to a BOS was used to test the pertinence of low dimensional models. These models were not built just by simplifying the physical processes involved: they were designed to capture the basic dynamical mechanisms behind the functioning of the syrinx and vocal tract. Specifically, the bifurcations leading to the vocalizations. Synthetic songs (and the models used to generate them) were considered pertinent as long as they were capable of eliciting physiological responses similar to those obtained using the BOS.

Besides the success of a simplified model to elicit responses, it is interesting to explore which physiological related parameters are necessary to include in the model (and tune) to obtain a physiological response. In this work we have discussed experiments in which noise and filter properties were changed, but a systematic study of responses as a function of the model’s parameters is an interesting program which is yet to be carried out. The nature of the response itself, and the way in which this degrades as these parameters are moved away from the optimal values is informative.

Low dimensional models can be used as synthesizers of acoustic stimuli, as a tool that allows to read physiological instructions in a behavioral key. In the past, muted birds could drive an electronic synthesizer implementing these dynamical models and sing (Arneodo et al., 2012). Beyond these applications, the construction of simplified models capable of eliciting responses in highly selective neurons in the auditory pathway highlights the existence of mechanisms, parametrized by a reasonably small number of parameters, which might help us order our studies of the extremely complex and rich field of vocal communication. As we have discussed, these low dimensional models have not emerged out of a unique leap of intuition: they are the result of a long and fascinating discussion that included musical analogies, careful analysis of sound, experimentation and theoretical work.

Notably, the vocal organs of different species of oscine birds are very similar, and the models described in this work are designed to describe song production by these species. But there are approximately other 6,000 bird species displaying a remarkable morphological diversity. We expect fascinating physics and dynamics to be at play in non-oscines. Departing from the specific problem of birdsong, we expect this integrative view to allow us to deepen our understanding of many other neuroethological problems.

## Author Contributions

Both authors wrote this review.

## Conflict of Interest

The authors declare that the research was conducted in the absence of any commercial or financial relationships that could be construed as a potential conflict of interest.

## References

[B1] Adachi S. Sato M. A. (1996). Trumpet sound simulation using a two-dimensional lip vibration model. *J. Acoust. Soc. Am.* 99 1200–1209. 10.1121/1.414601

[B2] Amador A. Margoliash D. (2011). “Auditory Memories and Feedback Processing for Vocal Learning,” in *The Auditory Cortex*, eds Winer J. A. Schreiner C. E. (Springer), 561–575. 10.1007/978-1-4419-0074-6_26

[B3] Amador A. Mindlin G. B. (2008). Beyond harmonic sounds in a simple model for birdsong production. *Chaos* 18:043123. 10.1063/1.3041023 19123633

[B4] Amador A. Mindlin G. B. (2014). Low dimensional dynamics in birdsong production. *Eur. Phys. J. B* 87 1–8.

[B5] Amador A. Sanz Perl Y. Mindlin G. B. Margoliash D. (2013). Elemental gesture dynamics are encoded by song premotor cortical neurons. *Nature* 495 59–64. 10.1038/nature11967 23446354 PMC3878432

[B6] Amit D. J. (1992). *Modeling brain function: The world of attractor neural networks.* Cambridge: Cambridge university press.

[B7] Arneodo E. M. Perl Y. S. Goller F. Mindlin G. B. (2012). Prosthetic avian vocal organ controlled by a freely behaving bird based on a low dimensional model of the biomechanical periphery. *PLoS Comput. Biol.* 8:e1002546. 10.1371/journal.pcbi.1002546 22761555 PMC3386162

[B8] Bolhuis J. J. Gahr M. (2006). Neural mechanisms of birdsong memory. *Nat. Rev. Neurosci.* 7 347–357. 10.1038/nrn.1904 16760915

[B9] Bush A. Doppler J. F. Goller F. Mindlin G. B. (2018). Syringeal EMGs and synthetic stimuli reveal a switch-like activation of the songbird’s vocal motor program. *Proc. Natl. Acad. Sci. U S A.* 115 8436–8441. 10.1073/pnas.1801251115 30068604 PMC6099895

[B10] Cardin J. A. Schmidt M. F. (2003). Song system auditory responses are stable and highly tuned during sedation, rapidly modulated and unselective during wakefulness, and suppressed by arousal. *J. Neurophysiol.* 90 2884–2899. 10.1152/jn.00391.2003 12878713

[B11] Cuvier G. (1800). Sur les instruments de la voix des oiseaux. *J. Physique Chimie d’Histoire Nat.* 50 426–451.

[B12] Dave A. S. Margoliash D. (2000). Song replay during sleep and computational rules for sensorimotor vocal learning. *Science* 290 812–816. 10.1126/science.290.5492.812 11052946

[B13] Doppler J. F. Bush A. Goller F. Mindlin G. B. (2018). From electromyographic activity to frequency modulation in zebra finch song. *J. Comp. Physiol. A Neuroethol. Sens. Neural. Behav. Physiol.* 204 209–217. 10.1007/s00359-017-1231-3 29170980

[B14] Doupe A. J. Konishi M. (1991). Song-selective auditory circuits in the vocal control system of the zebra finch. *Proc. Natl. Acad. Sci. U S A.* 88 11339–11343. 10.1073/pnas.88.24.11339 1763048 PMC53130

[B15] Elemans C. P. (2014). The singer and the song: The neuromechanics of avian sound production. *Curr. Opin. Neurobiol.* 28 172–178. 10.1016/j.conb.2014.07.022 25171107

[B16] Elemans C. P. Rasmussen J. H. Herbst C. T. During D. N. Zollinger S. A. Brumm H. (2015). Universal mechanisms of sound production and control in birds and mammals. *Nat. Commun.* 6:8978. 10.1038/ncomms9978 26612008 PMC4674827

[B17] Fletcher N. H. Riede T. Suthers R. A. (2006). Model for vocalization by a bird with distensible vocal cavity and open beak. *J. Acoust. Soc. Am.* 119 1005–1011. 10.1121/1.2159434 16521762

[B18] Gardner T. Cecchi G. Magnasco M. Laje R. Mindlin G. B. (2001). Simple motor gestures for birdsongs. *Phys. Rev. Lett.* 8720:208101. 10.1103/Physrevlett.87.208101 11690514

[B19] Goller F. Larsen O. N. (1997). A new mechanism of sound generation in songbirds. *Proc. Natl. Acad. Sci. U S A.* 94 14787–14791. 10.1073/pnas.94.26.14787 9405691 PMC25115

[B20] Goller F. Suthers R. A. (1996a). Role of syringeal muscles in controlling the phonology of bird song. *J. Neurophysiol.* 76 287–300. 10.1152/jn.1996.76.1.287 8836225

[B21] Goller F. Suthers R. A. (1996b). Role of syringeal muscles in gating airflow and sound production in singing brown thrashers. *J. Neurophysiol.* 75 867–876. 10.1152/jn.1996.75.2.867 8714659

[B22] Greenewalt C. H. (1968). *Bird song: Acoustics and physiology.* Washington: Smithsonian Institution Press.

[B23] Guckenheimer J. Holmes P. (1997). *Nonlinear oscillations, dynamical systems, and bifurcations of vector fields.* Berlin: Springer.

[B24] Herzel H. Berry D. Titze I. Steinecke I. (1995). Nonlinear dynamics of the voice: signal analysis and biomechanical modeling. *Chaos Interdiscipl. J. Nonline. Sci.* 5 30–34. 10.1063/1.166078 12780151

[B25] Howe M. S. (2003). *Theory of vortex sound.* Cambridge: Cambridge university press.

[B26] Ishizaka K. Flanagan J. L. (1972). Synthesis of voiced sounds from a two-mass model of the vocal cords. *Bell Syst. Technic. J.* 51 1233–1268. 10.1002/j.1538-7305.1972.tb02651.x

[B27] Janata P. Margoliash D. (1999). Gradual emergence of song selectivity in sensorimotor structures of the male zebra finch song system. *J. Neurosci.* 19 5108–5118. 10.1523/jneurosci.19-12-05108.1999 10366643 PMC6782639

[B28] Keener J. P. Sneyd J. (1998). *Mathematical physiology*, Vol. 1. New York NY: Springer.

[B29] Margoliash D. (1983). Acoustic parameters underlying the responses of song-specific neurons in the white-crowned sparrow. *J. Neurosci.* 3 1039–1057. 10.1523/jneurosci.03-05-01039.1983 6842281 PMC6564505

[B30] Margoliash D. Konishi M. (1985). Auditory representation of autogenous song in the song system of white-crowned sparrows. *Proc. Natl. Acad. Sci. U S A.* 82 5997–6000. 10.1073/pnas.82.17.5997 16593601 PMC390681

[B31] Mindlin G. B. (2017). Nonlinear dynamics in the study of birdsong. *Chaos* 27:092101. 10.1063/1.4986932 PMC5605333 28964148

[B32] Mindlin G. B. Laje R. (2005). *The physics of birdsong.* Berlin: Springer Verlag.

[B33] Mooney R. (2020). The neurobiology of innate and learned vocalizations in rodents and songbirds. *Curr. Opin. Neurobiol.* 64 24–31. 10.1016/j.conb.2020.01.004 32086177 PMC7431370

[B34] Nieder A. Mooney R. (2020). The neurobiology of innate, volitional and learned vocalizations in mammals and birds. *Philos. Trans. R Soc. Lond. B Biol. Sci.* 375:20190054. 10.1098/rstb.2019.0054 31735150 PMC6895551

[B35] Nottebohm F. Stokes T. M. Leonard C. M. (1976). Central control of song in the canary, Serinus canarius. *J. Comparat. Neurol.* 165 457–486. 10.1002/cne.901650405 1262540

[B36] Nowicki S. (1987). Vocal tract resonances in oscine bird sound production: evidence from bird- songs in a helium atmosphere. *Nature* 325 53–55. 10.1038/325053a0 3796738

[B37] Nowicki S. Marler P. (1988). How do birds sing? *Music Percept.* 5 391–426.

[B38] Olson H. F. (1967). *Music, physics and engineering.* New York, NY: Dover Publications, Inc.

[B39] Perl Y. S. Arneodo E. M. Amador A. Goller F. Mindlin G. B. (2011). Reconstruction of physiological instructions from Zebra finch song. *Physic. Rev. E* 84:051909. 10.1103/Physreve.84.051909 PMC3909473 22181446

[B40] Perl Y. S. Arneodo E. M. Amador A. Mindlin G. B. (2012). Nonlinear dynamics and the synthesis of Zebra finch song. *Int. J. Bifurcation Chaos* 22:1250235. 10.1142/s0218127412502355

[B41] Prather J. F. Peters S. Nowicki S. Mooney R. (2008). Precise auditory-vocal mirroring in neurons for learned vocal communication. *Nature* 451 305–U302. 10.1038/nature06492 18202651

[B42] Riede T. Goller F. (2010). Peripheral mechanisms for vocal production in birds - differences and similarities to human speech and singing. *Brain Lang.* 115 69–80. 10.1016/j.bandl.2009.11.003 20153887 PMC2896990

[B43] Riede T. Suthers R. A. Fletcher N. H. Blevins W. E. (2006). Songbirds tune their vocal tract to the fundamental frequency of their song. *Proc. Natl. Acad. Sci. U S A.* 103 5543–5548. 10.1073/Pnas.0601262103 16567614 PMC1459391

[B44] Shapiro M. B. Kenyon R. V. (2000). Control variables in mechanical muscle models: A mini-review and a new model. *Motor Control* 4 329–349. 10.1123/mcj.4.3.329 10970152

[B45] Sitt J. D. Amador A. Goller F. Mindlin G. B. (2008). Dynamical origin of spectrally rich vocalizations in birdsong. *Physic. Rev. E* 78:011905. 10.1103/PhysRevE.78.011905 18763980

[B46] Sitt J. D. Arneodo E. M. Goller F. Mindlin G. B. (2010). Physiologically driven avian vocal synthesizer. *Phys. Rev. E* 81:31927. 10.1103/PhysRevE.81.031927 20365790

[B47] Steinecke I. Herzel H. (1995). Bifurcations in an asymmetric vocal-fold model. *J. Acoust. Soc. Am.* 97 1874–1884. 10.1121/1.412061 7699169

[B48] Strogatz S. H. (1994). *Nonlinear dynamics and chaos: with applications to physics, biology, chemistry and engineering.* Massachusetts: Perseus Books.

[B49] Suthers R. A. (2001). Peripheral vocal mechanisms in birds: are songbirds special? *Netherl. J. Zool.* 51 217–242. 10.1163/156854201750385163

[B50] Suthers R. A. Goller F. Pytte C. (1999). The neuromuscular control of birdsong. *Philosop. Transact. R. Soc. Lond. Ser. B Biol. Sci.* 354 927–939. 10.1098/rstb.1999.0444 PMC1692586 10382225

[B51] Suthers R. A. Margoliash D. (2002). Motor control of birdsong. *Curr. Opin. Neurobiol.* 12 684–690. 10.1016/s0959-4388(02)00389-0 12490259

[B52] Thorpe W. H. (1959). Talking birds and the mode of action of the vocal apparatus of birds. *Proc. Zool. Soc.* 1 441–455. 10.1111/j.1469-7998.1959.tb05530.x

[B53] Titze I. R. (1988). The physics of small-amplitude oscillation of the vocal folds. *J. Acoust. Soc. Am.* 83 1536–1552. 10.1121/1.395910 3372869

[B54] Titze I. R. (1994). *Principles of voice production.* Englewood Cliffs, NJ: Prentice Hall.

[B55] Tytell E. D. Holmes P. Cohen A. H. (2011). Spikes alone do not behavior make: why neuroscience needs biomechanics. *Curr. Opin. Neurobiol.* 21 816–822. 10.1016/j.conb.2011.05.017 21683575 PMC3183174

[B56] Uribarri G. Rodriguez-Cajarville M. J. Tubaro P. L. Goller F. Mindlin G. B. (2020). Unusual Avian Vocal Mechanism Facilitates Encoding of Body Size. *Phys. Rev. Lett.* 124:098101. 10.1103/PhysRevLett.124.098101 32202899

[B57] Wild J. M. (2004). Functional neuroanatomy of the sensorimotor control of singing. *Behav. Neurobiol. Birdsong* 1016 438–462. 10.1196/Annals.1298.016 15313789

[B58] Young B. K. Mindlin G. B. Arneodo E. Goller F. (2017). Adult zebra finches rehearse highly variable song patterns during sleep. *PeerJ* 5:e4052. 10.7717/peerj.4052 29158983 PMC5694654

[B59] Zeigler H. P. Marler P. (2012). *Neuroscience of Birdsong.* Cambridge: Cambridge University Press.

[B60] Zhang Y. S. Ghazanfar A. A. (2018). Vocal development through morphological computation. *PLoS Biol.* 16:e2003933. 10.1371/journal.pbio.2003933 29462148 PMC5834215

[B61] Zhang Y. S. Takahashi D. Y. Liao D. A. Ghazanfar A. A. Elemans C. P. (2019). Vocal state change through laryngeal development. *Nat. Communicat.* 10 1–12. 10.1038/s41467-019-12588-6 PMC6785551 31597928

